# Laparoscopic Splenectomy for Splenic Artery Aneurysms Associated With Infective Endocarditis: A Case Report

**DOI:** 10.7759/cureus.66740

**Published:** 2024-08-12

**Authors:** Kei Harada, Yuichiro Kawamura, Keiji Nagata, Takahisa Fujikawa

**Affiliations:** 1 Surgery, Kokura Memorial Hospital, Kitakyushu, JPN

**Keywords:** infective endocarditis, splenic artery aneurysm, visceral artery aneurysm, laparoscopic splenectomy, splenectomy

## Abstract

Splenic artery aneurysms (SAAs) are a relatively uncommon but potentially life-threatening disease. In recent years, although there have been an increasing number of reports of interventional radiology (IVR) treatment for SAAs, there are still many cases in which surgical intervention is required. In particular, SAAs associated with infective endocarditis (SAAs-IE) are rare, and the treatment strategies and perspectives for SAAs-IE remain controversial. Herein, we report a successful case of laparoscopic splenectomy for SAAs-IE with a literature review.

## Introduction

Splenic artery aneurysms (SAAs) are a rare but potentially life-threatening condition, and the incidence of SAAs in the general population ranges from 0.1% to 10.4% [1,2]. SAAs and other visceral artery aneurysms (VAAs) are being diagnosed with increasing frequency, including as incidental findings, with the use of advanced imaging techniques [3].

In recent years, interventional radiology (IVR) has become the primary treatment for SAAs, but there are still many cases that require surgical intervention [3,4]. Conventional surgery for SAAs includes proximal and distal ligation and aneurysmectomy for lesions in the proximal or mid-splenic artery [3-5]. However, SAAs adjacent to the splenic hilum and infected SAAs, as in our case, often require splenectomy [4,6]. Furthermore, there are an increasing number of reports of treating SAAs through surgical procedures, such as laparoscopic splenectomy, hand-assisted laparoscopic surgery (HALS), and robot-assisted splenectomy [7-9].

Although there are several reports on treatment strategies for SAAs, treatment strategies and perspectives for SAAs associated with infective endocarditis (SAAs-IE) are controversial [6,10]. Herein, we report a successful case of laparoscopic splenectomy for SAAs-IE and suggest that this may be helpful in considering treatment for SAAs-IE.

## Case presentation

**Figure 1:**
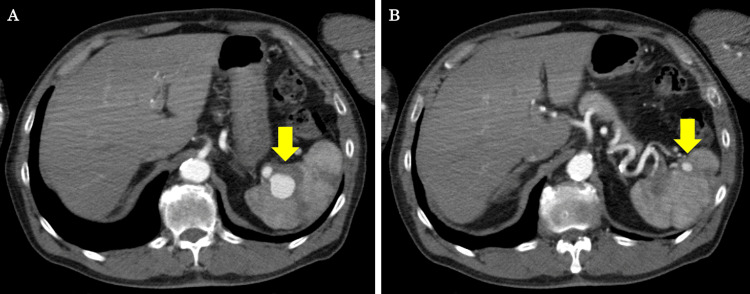
CECT findings. (A) A 25-mm dilated aneurysm was found at the distal end of the upper pole branch of the splenic artery (a yellow arrow). (B) A 10-mm aneurysm was found at the end of the lower pole branch of the splenic artery (a yellow arrow). CECT: contrast-enhanced computed tomography

A 70-year-old man was referred to the department of internal medicine with the chief complaints of fever, anorexia, and fatigue for the past 2-3 days. Auscultation revealed a heart murmur, and transthoracic echocardiography (TTE) revealed severe aortic regurgitation (AR) and severe mitral regurgitation (MR), as well as perforation of the aortic and mitral valves. These test results led to a diagnosis of healed infective endocarditis (healed-IE). Coronary angiography (CAG) also revealed coronary artery stenosis, and aortic valve replacement (AVR), mitral valve replacement (MVR), and coronary artery bypass grafting (CABG) were performed in the cardiovascular surgery department. The postoperative course was uneventful, and the patient was discharged on postoperative day (POD) 32. Then, four months later, a follow-up contrast-enhanced computed tomography (CECT) incidentally revealed SAAs, including a 25-mm splenic aneurysm at the splenic hilum (Figure 1A-1B), and the patient was referred to our surgical department for further examination and treatment.

**Figure 2:**
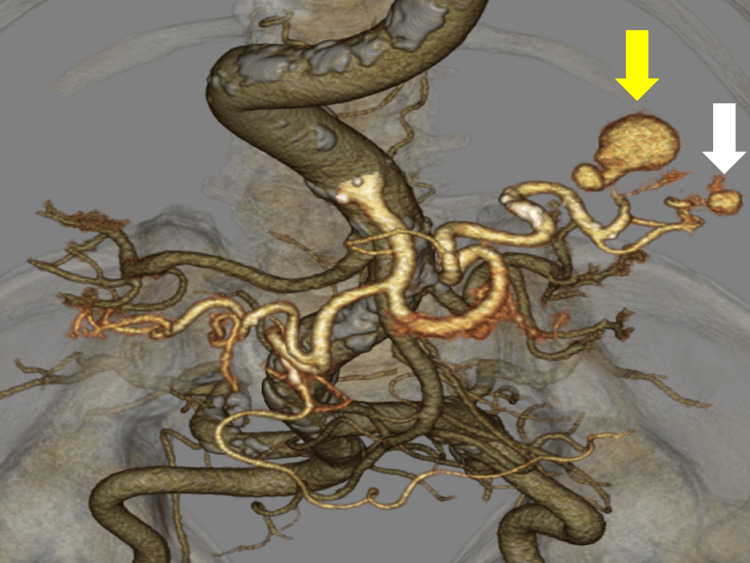
3D vascular reconstruction image findings. A dilated aneurysm was found at the distal end of the upper pole branch of the splenic artery (a yellow arrow) and an aneurysm at the end of the lower pole branch (a white arrow). 3D: three-dimensional

At that time, the patient was hemodynamically stable and afebrile. On physical examination, his abdomen was soft with no tenderness, distention, or peritoneal symptoms. His laboratory values were a C-reactive protein (CRP) level of 0.2 mg/dL, a white blood cell (WBC) level of 8300/μl, and a fibrin degradation product D-dimer level of 1.3 μg/L. The liver and renal function tests were within the normal limits. We used computed tomography angiography (CTA) to obtain detailed images of the patient's vascular system, followed by three-dimensional (3D) volume rendering for enhanced visualization. A 3D vascular reconstruction image showed an aneurysm approximately 25 mm in size at the distal end of the upper pole branch of the splenic artery and an aneurysm approximately 10 mm in size at the distal end of the lower pole branch (Figure 2).

These SAAs had suddenly appeared, although they had not been present at the time of the most recent onset of IE, making the possibility of SAAs-IE high. In addition, because there were multiple SAAs and they were located in the splenic hilum, treatment with IVR carried a high risk of splenic infarction due to organ ischemia. Because aneurysm resection and anastomosis by a vascular surgeon would also be anatomically difficult, we consulted with a radiologist and a vascular surgeon and decided to perform a laparoscopic splenectomy, including the aneurysms. Before performing the operation for the SAAs, we performed a systemic examination to confirm that no aneurysms had appeared in blood vessels other than the splenic artery.

**Figure 3:**
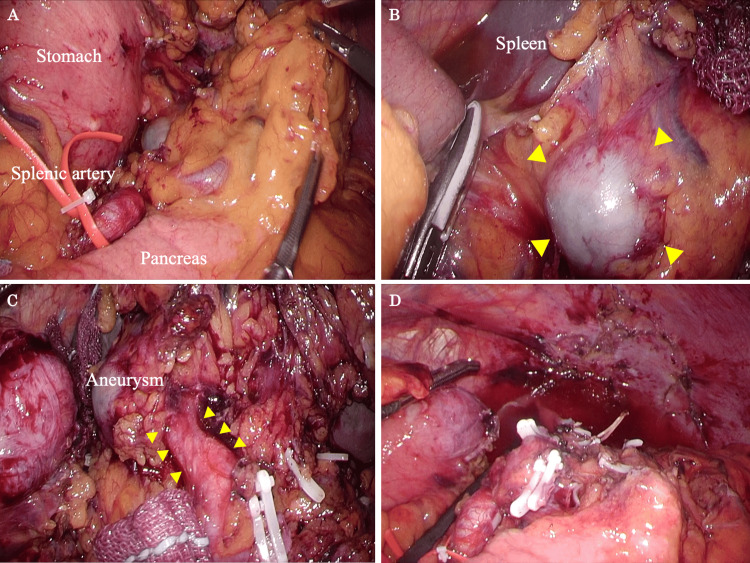
Main surgical procedures for laparoscopic splenectomy. (A) The splenic artery trunk is taped and the anatomy around the spleen is confirmed. (B) A dilated aneurysm was observed (yellow arrows), and mobilization of the upper pole of the spleen was performed. (C) After processing the other vessels, the vessels leading to the dilated aneurysm are identified (yellow arrows). (D) After splenectomy.

The surgical findings and procedures are presented here. The splenic artery trunk is taped so that bleeding can be controlled if it occurs suddenly during the dissection procedure at the splenic hilum (Figure 3A). In this case, a dilated aneurysm was observed in the upper pole, so mobilization of the spleen was kept to a minimum (Figure 3B). The pancreas was slightly separated, and the splenic lower pole branch, the pancreatic tail artery, and the splenic vein were clipped and dissected (Figure 3C). The upper pole branch was identified, and a dilated aneurysm was confirmed at its distal end, which was then clipped and dissected. After all vessels were dissected, the spleen, which included SAAs, was removed, and the operation was over (Figure 3D).

**Figure 4:**
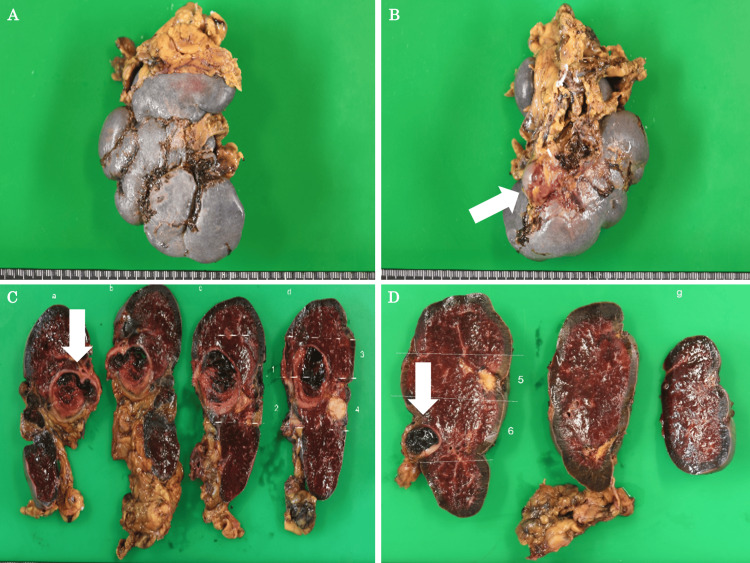
The resected spleen specimen. (A, B) The resected spleen was 9.7 × 7.9 × 4.6 cm in size, and a 30-mm aneurysm was found at the splenic hilum (a white arrow). (C) A 30-mm aneurysm was found. (D) A 10-mm aneurysm was found.

The operative time was five hours and 42 minutes, and 320 ml of blood was lost. There were no significant perioperative complications, and the patient was discharged on POD 9. Although we confirmed that the patient had received the pneumococcal vaccine before the surgery, we followed up closely after the patient was discharged from the hospital because of the possibility of developing a serious infection after splenectomy. As a result, the patient has progressed without any serious infections or significant changes in platelet counts since being discharged from the hospital. The resected spleen was 9.7 × 7.9 × 4.6 cm in size, and a 30-mm aneurysm and a 10-mm aneurysm were found at the splenic hilum (Figure 4A-4D).

**Figure 5:**
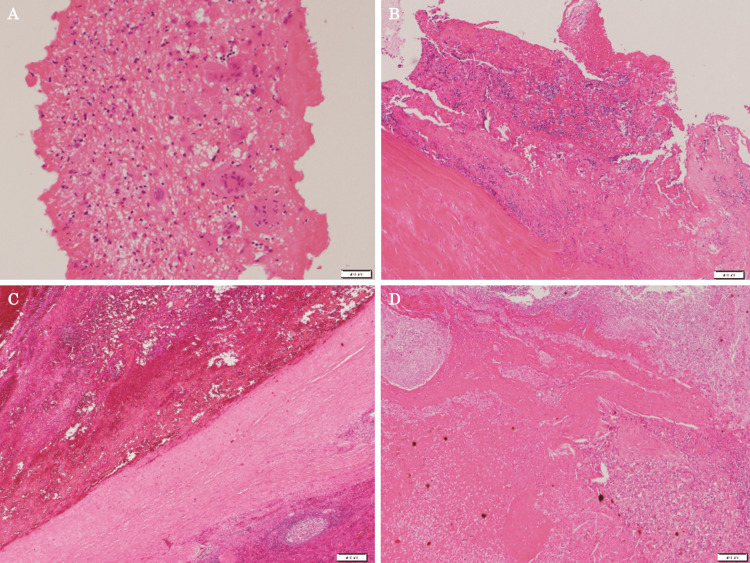
The pathological findings of the resected specimen. (A) The resected aortic valve tissue with myxoid degeneration, hyalinization, calcification, and granulation tissue. (B) The resected mitral valve tissue with hyalinized and granulated tissue. (C) The wall of the resected splenic aneurysm. (D) The area of ​​splenic infarction.

Histologically, it was an aneurysm with fibrin and an organized thrombus inside, and the white area on the cut surface was infarct necrosis. Small amounts of epithelioid cell granulomas were seen around it. Both the aortic valve and mitral valve that had been removed in the previous surgery were tissues with hyalinization, calcification, and granulation tissue, and fibrin with lymphocytes and multinucleated giant cells was also seen. Although no bacterial masses were seen, the pathological findings of this case were consistent with SAAs-IE from a histological point of view (Figure 5A-5D). In reporting the case, we respected ethical considerations, including the protection of personal privacy, and provided sufficient informed consent to the patient.

## Discussion

VAAs are a rare but possibly life-threatening condition [11,12]. Some studies based on autopsy have reported a prevalence of VAAs as high as 10% [11,12]. This is attributable to the recent spread of noninvasive imaging diagnostics as well as a rise in the number of VAAs discovered incidentally during CT scans [3]. Among VAAs, SAAs are common, accounting for 60-80% [13,14]. Considering the general population, the incidence of SAAs has been reported to range from 0.1% to 10.4% [1,2]. Although the pathophysiology of SAAs is still not fully understood, trauma, hormonal and local hemodynamic events during pregnancy, portal hypertension, arterial degeneration, and atherosclerosis have all been implicated [5,11,14]. In recent years, various minimally invasive techniques have been developed for the treatment of SAAs, including laparoscopic surgery, endovascular embolization, and aneurysm removal using stent grafts, and treatment outcomes have improved [15]. Among minimally invasive treatments, the choice of surgery must be determined by the location of the SAAs, the surgeon's experience, and the urgency of the intervention (emergency or elective).

As in our case, SAAs at the splenic hilum are one of the SAAs for which surgical intervention is required [4]. Distal SAAs close to the splenic hilum should not be treated with IVR because they can cause splenic infarction and pancreatitis due to end-organ ischemia [4,13]. Furthermore, multiple SAAs were present in our case, which is considered to increase the risk of splenic infarction as a result of IVR [5]. Also, an important factor in our decision to perform surgical intervention in this case was the SAAs-IE. An infected aneurysm is a localized artery dilation that happens when an infection damages the arterial wall [1]. Although SAAs-IE are a rare occurrence that may be clinically silent, they can quickly grow and rupture, which can lead to a number of complications [6,10]. Even if there are no CT findings characteristic of infectious aneurysms, such as fluid accumulation around the aneurysm or air around the aneurysm, a rapidly expanding aneurysm on a follow-up CT scan is suggestive of an infectious aneurysm [10]. Although there is no gold standard treatment strategy for SAAs-IE, early treatment, such as surgery or IVR, and strict follow-up after treatment are necessary to avoid serious complications [10,13].

Laparoscopic splenectomy for SAAs has been reported to have better outcomes than traditional open splenectomy [8,9]. The benefit of laparoscopic splenectomy is that it uses a flexible scope to enable clear sight of the upper pole and dorsal side of the spleen, which is challenging to view during open surgery. It is also possible to recognize SAAs and the branching of small blood vessels because of the magnifying effect. The reasons we decided to perform laparoscopic splenectomy in our case were because the splenic volume was typical and there were no varices [4]. Fortunately, the inflammation around the SAAs was mild, and there was an anatomical distance between the SAAs and the body and tail of the pancreas, so distal pancreatectomy was not performed. We believe this was due to the success of antibiotic treatment and the fact that the surgery could be performed electively. In addition, there was no transition to HALS. The reason for treating the vessels of the splenic hilar trunk individually without using an automatic suture machine was to avoid damaging the SAAs with the cartridge.

## Conclusions

We experienced a successful case in which laparoscopic splenectomy was performed for SAAs-IE. This report suggests that, under appropriate conditions, laparoscopic splenectomy after suppression of inflammation with antibiotic therapy is a preferable option. SAAs, as in our case, still require strict follow-up, and effective treatment strategies should be developed as the number of cases continues to increase.

## References

[1] Dave SP, Reis ED, Hossain A, Taub PJ, Kerstein MD, Hollier LH (2000). Splenic artery aneurysm in the 1990s. Ann Vasc Surg.

[2] Al-Habbal Y, Christophi C, Muralidharan V (2010). Aneurysms of the splenic artery - a review. Surgeon.

[3] Chaer RA, Abularrage CJ, Coleman DM, Eslami MH, Kashyap VS, Rockman C, Murad MH (2020). The Society for Vascular Surgery clinical practice guidelines on the management of visceral aneurysms. J Vasc Surg.

[4] Pulli R, Dorigo W, Troisi N, Pratesi G, Innocenti AA, Pratesi C (2008). Surgical treatment of visceral artery aneurysms: a 25-year experience. J Vasc Surg.

[5] Stanley JC, Wakefield TW, Graham LM, Whitehouse WM, Zelenock GB, Lindenauer SM (1986). Clinical importance and management of splanchnic artery aneurysms. J Vasc Surg.

[6] Boukobza M, Raffoul R, Rebibo L, Khalil A, Laissy JP (2024). Splenic artery infectious aneurysms in infective endocarditis - an observational study and comprehensive literature review. Ann Vasc Surg.

[7] Suzuki H, Shimura T, Asao T (2002). Laparoscopic resection of splenic artery aneurysm; a case report. Hepatogastroenterology.

[8] Nasser HA, Kansoun AH, Sleiman YA (2018). Different laparoscopic treatment modalities for splenic artery aneurysms: about 3 cases with review of the literature. Acta Chir Belg.

[9] Małczak P, Wysocki M, Major P (2017). Laparoscopic approach to splenic aneurysms. Vascular.

[10] McCready RA, Bryant MA, Fehrenbacher JW, Rowe MG (2007). Infected splenic artery aneurysm with associated splenic abscess formation secondary to bacterial endocarditis: case report and review of the literature. J Vasc Surg.

[11] Corey MR, Ergul EA, Cambria RP (2016). The natural history of splanchnic artery aneurysms and outcomes after operative intervention. J Vasc Surg.

[12] Tessier DJ, Stone WM, Fowl RJ, Abbas MA, Andrews JC, Bower TC, Gloviczki P (2003). Clinical features and management of splenic artery pseudoaneurysm: case series and cumulative review of literature. J Vasc Surg.

[13] Berceli SA (2005). Hepatic and splenic artery aneurysms. Semin Vasc Surg.

[14] Hosn MA, Xu J, Sharafuddin M, Corson JD (2019). Visceral artery aneurysms: decision making and treatment options in the new era of minimally invasive and endovascular surgery. Int J Angiol.

[15] Ouchi T, Kato N, Nakajima K, Higashigawa T, Hashimoto T, Chino S, Sakuma H (2018). Splenic artery aneurysm treated with endovascular stent grafting: a case report and review of literature. Vasc Endovascular Surg.

